# MicroRNAs and DNA-Damaging Drugs in Breast Cancer: Strength in Numbers

**DOI:** 10.3389/fonc.2018.00352

**Published:** 2018-09-03

**Authors:** Ilaria Plantamura, Giulia Cosentino, Alessandra Cataldo

**Affiliations:** Molecular Targeting Unit, Research Department, Fondazione IRCCS Istituto Nazionale dei Tumori, Milan, Italy

**Keywords:** breast cancer, DNA repair, DNA damage response, DNA-damaging drugs, microRNAs

## Abstract

MicroRNAs are a class of small non-coding regulatory RNAs playing key roles in cancer. Breast cancer is the most common female malignancy worldwide and is categorized into four molecular subtypes: luminal A and B, HER2+ and triple-negative breast cancer (TNBC). Despite the development of multiple targeted therapies for luminal and HER2+ breast tumors, TNBC lacks specific therapeutic approaches, thus they are treated mainly with radio- and chemotherapy. The effectiveness of these therapeutic regimens is based on their ability to induce DNA damage, which is differentially resolved and repaired by normal vs. cancer cells. Recently, drugs directly targeting DNA repair mechanisms, such as PARP inhibitors, have emerged as attractive candidates for the future molecular targeted-therapy in breast cancer. These compounds prevent cancer cells to appropriate repair DNA double strand breaks and induce a phenomenon called *synthetic lethality*, that results from the concurrent inhibition of PARP and the absence of functional BRCA genes which prompt cell death. MicroRNAs are relevant players in most of the biological processes including DNA damage repair mechanisms. Consistently, the downregulation of DNA repair genes by miRNAs have been probe to improve the therapeutic effect of genotoxic drugs. In this review, we discuss how microRNAs can sensitize cancer cells to DNA-damaging drugs, through the regulation of DNA repair genes, and examine the most recent findings on their possible use as a therapeutic tools of treatment response in breast cancer.

## General overview

### MicroRNAs

MicroRNAs (miRNAs) are endogenous, small non-coding RNAs that regulate gene expression at post-transcriptional level. Mature miRNAs are single strand molecules of ~18–25 nucleotides (nt), transcribed by RNA polymerase II/III as long primary transcripts with a hairpin structure, called pri-miRNAs. Pri-miRNAs are then cleaved in the nucleus into ~60 nt long molecules (pre-miRNAs) by the Microprocessor, a multi-protein complex comprising the RNase III enzyme DROSHA and its cofactor DGCR8 (1, 2). As pre-miRNAs, these molecules are specifically recognized by the nuclear export machinery, mainly composed of Exportin-5 and Ran-GTPase, and exported to the cytoplasm where their processing is completed. The dsRNA stem of pre-miRNAs is asymmetrically cleaved by the second multi-domain RNAase III enzyme DICER into a short nucleotide duplex (3). During this step, the transactivation-responsive RNA-binding protein (TRBP) mediates the assembling of the miRNA-induced silencing complex (miRISC), favoring DICER and Argonaute protein (AGO1, AGO2, AGO3 or AGO4) interaction (4). The miRISC complex selects the mature miRNA (guide strand), which then guides the machinery to the target mRNA (5). MiRNA/mRNA interaction occurs by the recognition between the “seed” region at the miRNA 5′UTR and its complementary sequence on the 3′UTR of the designated mRNA. The result of the pairing is, either the translational repression or transcript degradation, in dependence of the complementary degree between the two sequences (6). After the discovery of the small RNA lin-4 function in the larval development of *Caenorhabditis elegans* in 1993, many researchers had started to investigate the regulatory potential of these small molecules (7). To date, it is well-known that miRNAs participate in almost every biological process in mammals, including cancer (8). Several mechanisms alter miRNA expression in cancer such as genomic aberrations, epigenetic changes, dysfunction of the processing machinery, alteration of transcription factor expression, among others (9).

In cancer, miRNAs can act as tumor suppressors or oncogenes (oncomiR). Functionally, miRNAs with a tumor suppressor role target oncogenes and are generally downregulated in cancer cells (e.g., miR-205 and miR-34 in breast cancer). While, oncogenic miRNAs target tumor suppressor genes and are usually upregulated in tumor cells (e.g., miR-21, miR-155, and miR-221/222 in breast cancer) (10).

MiRNA capability to regulate several target genes involved in oncogenic mechanism such as proliferation, progression, metastasis, and therapy response, makes these small molecules fascinating candidates as therapeutic tools. In fact, recent studies have been focused on develop new strategies to make possible the miRNA-based therapy approach. Generally, miRNAs can be reintroduced in cancer cells using miRNA *mimics* or be inhibited by *anti-miRs*. During the last years, new methods to deliver and to stabilize miRNA *mimics* and *anti-miRs* have been developed, some of which are currently in clinical trials.

MiRNA *mimics* and *anti-miRs* can be delivered with lipid carriers, for instance the miR-34-based therapy MRX34 (Mirna Therapeutics) deliver miR-34 *mimic* sequence through the lipid carrier NOV40. MRX34 is the first miRNA-based therapy undergoing in a clinical trial for cancer treatment. During 2013, patients with lymphoma, melanoma, multiple myeloma and liver, small cell lung and renal carcinoma were enrolled in a phase I clinical trial. Unfortunately, despite the promising results obtained with the partial response of 3 patients and stable disease in other 14 patients, the trial was terminated in September 2016 due to severe and lethal immune-related adverse reactions occurred in some patients (clinicaltrials.gov:NCT01829971) (11, 12). Additionally, EnGeneIC Delivery Vehicle (EDV) nanocells (also called TargomiRs) coated with epidermal growth factor receptor (EGFR)-specific antibodies are currently in a phase I trial to deliver miR-16 mimics in patients with malignant pleural mesothelioma and NSCLC (clinicaltrials.gov: NCT02369198), current preliminary results show that the treatment is well-tolerated. MiRNA *mimics* can be also conjugated to *N*-acetyl-D-galactosamine (GalNAc) particles, improving their uptake into cells through clathrin-mediated endocytosis. Moreover, RG-125 (Regulus Therapeutics), a GalNAc-conjugated containing an *anti-miR*-103/107 sequence, recently entered in clinical investigations to treat non-alcoholic steatohepatitis (NASH). In addition, a phase I trial in HCV-infected patients was initiated to evaluate the response of RG-101 (Regulus Therapeutics), a *N*-acetyl-D-galactosamine (GalNAc)-conjugated *anti-miR* targeting miR-122. Finally, in November 2015, a LNA-modified anti-miR-155 (MRG-106) has begun to test in a phase I clinical trial to treat patients with cutaneous T-cell lymphoma (clinicaltrials.gov: NCT02580552).

### Breast cancer

Breast cancer represents one of the most common malignancies worldwide and a leading cause of cancer-related death in women (13). Biological and genomic characterizations have described breast cancer as a highly heterogeneous disease, according to histological and molecular features, and responsiveness to therapy (14).

Clinically, breast cancers are firstly categorized, according to the expression of three receptors routinely assessed by immunohistochemistry assay in the following subtypes: estrogen and progesterone receptor positive (ER+, PR+), human epidermal growth factor receptor positive (HER2+) and triple-negative (ER–, PR–, HER2–) malignancies (15, 16). This classification provide valuable clinical information, mainly to choice the first line treatment, in addition to the histological grade, clinical stage, patient's age and menopausal status. The advent of high throughput technologies, such as microarray-based transcriptomic analysis, has provided new sources of information regarding breast cancer biology. Gene expression profiling of breast cancers identified five intrinsic molecular subtypes: hormone receptors positive luminal A and luminal B, HER2-enriched, basal-like and normal-like. These subtypes differ in incidence, prognosis and responsiveness to therapy (17–19). Luminal B (ER+, PR+, HER2–, Ki67+) usually present higher clinical grade than luminal A (ER+, PR+, HER2–, Ki67–) tumors, and some of them also express HER2 receptor. The HER2-enriched (HER2+, ER–, PR–) most frequently present high grade and node positive, whereas the basal-like (HER2–, ER–, PR–) subgroup mainly comprises triple-negative breast cancers (TNBCs) and frequently shows BRCA1 mutations, both germinal and sporadic (20).

An additional intrinsic subtype described more recently is the claudin-low subtype, sorted mainly from the TNBC subgroup and characterized by stem cell-like features (21, 22). A more detail classification based on molecular portrait of TNBCs have been provided in 2011 by Lehmann and colleagues through the identification of six subtypes with distinct gene expression patterns and response to treatment: basal-like 1 (BL1), basal-like 2 (BL2), immunomodulatory (IM), mesenchymal (M), mesenchymal stem-like (MSL), and luminal androgen receptor (LAR) subtype (23). High level of cell cycle and DNA damage response genes are expressed by BL1 and BL2 subtypes, which are preferentially sensitive to cisplatin. Epithelial-to-mesenchymal transition and growth factor pathways are enriched in the M and MSL subtypes, which respond to PI3K/mTOR and abl/src inhibitors. The LAR subtype, characterized by androgen receptor (AR) signaling and shorter relapse-free survival, is sensitive to bicalutamide (an AR antagonist). During the last years, next generation sequencing (NGS) has significantly improved the molecular characterization of breast carcinomas, providing data on gene mutations, DNA copy number variations, DNA methylation and miRNA expression patterns. Important examples of such studies are The Cancer Genome Atlas (TCGA) project, the International Cancer Genome Consortium (ICGC) and the Molecular Taxonomy of Breast Cancer International Consortium (METABRIC) (24, 25). Information gathered from these studies are particularly useful to the clinical practice, because they provide a list of new molecules, which can be potentially targeted or exploited for therapy interventions to improve drug efficacy (26).

The role of miRNAs in breast cancer was deeply investigated in the last decades. The first miRNA signature in breast cancer was described by Iorio et al. (27), followed by several studies that have demonstrated a functional role of miRNAs in the disease. One of the most studied miRNA in breast cancer is miR-21, which acts as an oncomiR mediating cell survival, proliferation, invasion, and metastasis (28). MiR-9, targeting E-cadherin and regulating the EMT process, is recognized as a metastamiR in breast cancer (29); as well as miR-10b, one of the first metastamiRs described in a breast cancer model (30). Conversely, many miRNAs have been identified as tumor suppressors in breast cancer. MiR-205 and miR-125a, for example, modulate the expression of HER3 and HER2 oncogenes, respectively (31, 32). Moreover, it has been reported that miR-205 and miR-200 family have an important anti-tumorigenic role by targeting ZEB1 and ZEB2, suppressing EMT process (33).

### DNA repair mechanisms

**Figure 1:**
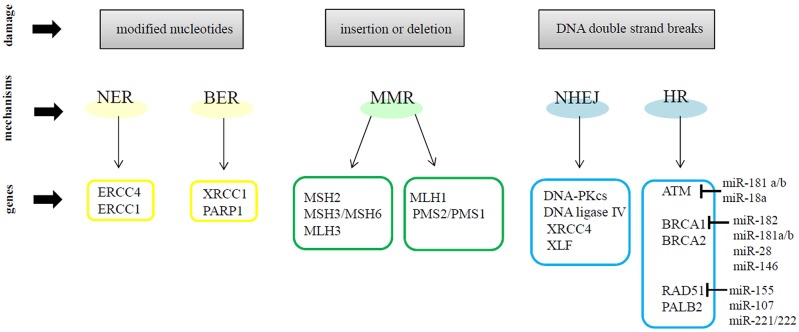
A schematic representation of DNA repair mechanisms and some relevant miRNAs involved in the modulation of DNA repair genes.

Genomic instability is well-recognized as one of the hallmarks of cancer (34). Many studies have demonstrated that breast cancer cells have defective DNA damage response (DDR) mechanisms. In general, when DNA damage occurs, cells repair the errors and continue to proliferate; otherwise, the damage can cause mutations or chromosomal rearrangements, which induce tumorigenesis. The DDR is a complex system activated upon DNA damage, shaped by the activity of DNA damage signal transduction, DNA repair mechanisms, cell cycle checkpoints and apoptosis signaling pathways (35). DDR regulates DNA repair by inducing the following molecular processes: detection of damage sites, recruitment of repair factors and repair of DNA lesions. DDR machinery is divided into DNA damage sensors, signal transducers and effectors. DDR can use different mechanisms to repair DNA damage. In particular, two mechanisms are designated to remove damaged and modified nucleotides: (1) Nucleotide Excision Repair (NER) which works on helix-distorting and transcription-blocking lesions (i.e., UV-induced pyrimidine dimers) and (2) Base Excision Repair (BER) which removes single nucleotides by methylation, alkylation, deamination, or oxidation (36, 37). Other DDR mechanism participates in the recognition of incorrect insertions or deletions of nucleotides during DNA synthesis, which can lead to microsatellite instability and cancer. These errors affect the canonic DNA sequence and induce base mismatches that cause the distortion of DNA helix. The Mismatch Repair Machinery (MMR) operates through MSH2 and MLH1, which form heterodimers with MSH3 or MSH6 and MLH3, PMS2, or PMS1, respectively (38, 39). Many environmental agents can cause other types of DNA damage such as: Double-Strand Breaks (DSBs) or Single-Strand Breaks (SSBs) (40). SSBs and modified bases are the most common DNA damage, approximately in 1 day occurs 20,000 events per cell, but are usually repaired via BER mechanism (41). Instead, DSBs induce the recruitment of MRE11–RAD50–NBS1 (MRN) complex, which activates the serine/threonine-specific kinases ATM which allows its auto-phosphorylation (pATM) and the phosphorylation of the Ser139 of histone H2AX (γH2AX) in response to DNA damage signals. γH2AX recruits additional pATM molecules and DDR proteins, such as p53-binding protein 1 (53BP1), at DSB site, to generate a nuclear foci (42, 43). In response to DSBs, ATM kinase promotes the phosphorylation of many proteins, in particular Chk2 kinase, one of the most important effectors of ATM (44, 45). On the other hand, ATR recruit Chk1 kinase after stalled replication-forks and SSBs induced by UV (46, 47). ATM and ATR induce the phosphorylation of multiple proteins to activate downstream DNA repair pathways and induce cell cycle arrest, apoptosis or senescence when damage repair was not efficient (48). DSBs are repaired by two mechanisms: (1) Non-Homologous End Joining (NHEJ), active during all phases of the cell cycle but its activation occur mainly in G0/G1 phases, or (2) Homologous Recombination (HR), which acts in the S/G2 phases of the cell cycle (49, 50). In particular, NHEJ resolve double-stranded DNA breaks by enhancing Ku protein binding, as well as the recruitment and activation of DNA-PK. NHEJ complex includes DNA ligase IV, XRCC4, and XLF/Cernunnos protein which promotes a direct ligation of the ends of DSBs. However, this mechanism can induce some alterations, such as deletions or mutations of DNA sequences at the DSB site or around it (40). HR comprehends a set of proteins, including BRCA1, BRCA2, RAD51, and PALB2 that allow the restoration of the original DNA sequence at the damage site. Briefly, the DNA sequence near to DSB is deleted and the sequence on the homologous sister chromatid is used as a template to synthesize new DNA at the DSB site (37, 49). Many studies have demonstrated that miRNAs regulate, at transcriptional and post-transcriptional levels, the DNA damage sensor, signal transducer and effector genes in cancer cells. For example, miR-182, miR-181a/b, miR-28, and miR-146 have been demonstrated to target BRCA1 in breast cancer cells. The DDR gene RAD51 is modulated by miR-155, miR-107, miR-221/222; whereas ATM is targeted by miR-181a/b and miR-18a. Moreover, in breast cancer cells, miR-125b and miR-34a are able to control the expression of TP53, the main cell cycle regulator (51). Figure 1 shows a schematic representation of DNA damage repair mechanisms and some relevant miRNAs involved in the modulation of DNA repair genes. Therefore, miRNAs represent an important regulatory mechanism of DNA repair pathways and a novel source to exploit DDR gene/miRNA interactions for clinical purposes as potential biomarkers and therapeutic tools.

In the next sections, we will review how miRNAs could influence the response to DNA-damaging drugs in breast cancer therapy. Indeed, several studies have reported a key role of miRNAs in the responsiveness to DNA-damage based therapies, by modulating the expression of genes involved in the initiation, activation and maintenance of DDR mechanisms.

## DNA damaging drugs in breast cancer therapy

The strategies of breast cancer treatment include surgery, radiation, chemotherapy, hormone therapy, and biological targeted therapy. Patients with hormone receptor positive (ER+, PR+) tumors receive hormone therapy (i.e., tamoxifen and aromatase inhibitor), whereas patients affected by HER2+ tumors receive anti-HER2 targeted therapy (i.e., trastuzumab and pertuzumab) (52). Thus, the biggest challenge is represented by the clinical management of TNBCs, mainly due to the lack of targeted drugs. Indeed, the standard therapy for these tumors still remains cytotoxic chemotherapy (53). In term of response to chemotherapy, luminal A tumors show lower responsiveness, luminal B are more responsive than luminal A but less than HER2-enriched and basal-like, which are the subgroups with the higher response rate.

In breast cancer management, radio- and chemotherapy exert their effects by causing DNA damage, and are usually used as first-line drugs in combination with hormone and target therapies. Ionizing radiation (IR), anthracyclines, platinum compounds, and taxanes usually induce DSBs and SSBs, the efficacy of current DNA-damaging drugs is correlated with the capabilities of cancer cells to resolve and repair DNA lesions. Cancer cells are highly proliferative in comparison with normal cells, this feature increases their susceptibility to DNA damage exposure in the S phase of the cell cycle (20). However, the main problem of radio- and chemotherapy is the development of acquired resistance along the drug administration.

Radiotherapy treatment, based on the administration of a specific amount of energy, induces the activation of multi-staged processes which enhance tumor cell death. In particular, DSBs promote chromosomal alterations and affect cell division, contributing to cell death or mutation (54). Ionizing radiation (IR), such as X-rays, can extend cell damage by direct DNA breaks or indirectly through the creation of free radicals (55, 56). Tumors receiving a large total dose of radiations sometimes develop radioresistance, which eventually leads to treatment failure. The acquired radioresistance can be associated with altered expression of cell cycle molecules and DDR effectors, such as the overexpression of cyclin D1 and the constitutive activation of DNA-PK and AKT, respectively (57).

Chemotherapy is the most common therapy for cancer. Chemotherapeutic agents promote tumor cell death by direct cytotoxicity, activation of host immune response, inhibition of cell proliferation and induction of apoptosis (58). After cytotoxic agents administration, DNA damage is the first event sensed by the cellular stress response machinery, triggering the activation of effector systems, such as apoptosis (59). Unfortunately, resistance to chemotherapy can occur at many levels including DNA repair, cell cycle regulation and evasion of apoptosis (60).

Recently, the identification of BRCA-associated DNA repair mechanisms, frequently impaired in TNBCs, led to the development of specific DNA damage target therapies.

BRCA genes, involved in DNA repair through HR after DSBs, are altered in sporadic and hereditary breast cancer; notably, in sporadic tumors BRCA1 mutations are rare (<5%) while ~10% of TNBC patients present germline mutations in BRCA1/2 which increase breast cancer risk about 60–70% (61, 62). In the last years, many groups have deep investigated the role of defective HR mechanisms in cancer: the so-called “BRCAness” status is defined as the presence in tumor cells of alternative mechanisms impairing BRCA1/2 functions, or the alteration in HR genes. BRCAness is a phenocopy of BRCA1/2 mutations, in fact, HR mechanisms result defective although tumor cells do not carry mutations in BRCA1/2 genes (63).

As above clarified, for patients affected by TNBCs, targeted therapies are not currently available and chemotherapy may lead the acquisition of resistance in the later stages of the disease (64). In the last years PARP inhibitors have emerged as a possible therapeutic approach, especially when cancer cells lack functional alleles of the genes BRCA1 or BRCA2 (65). Poly-[ADP-Ribose]-Polymerase-1 (PARP-1) is a crucial molecule involved in the activation of the DNA-damage response. PARP-1 is a nuclear protein implicated in various processes involving DNA-related transactions. PARP-1 recognizes DNA-damage sites and creates long chains of poly-ADP-ribose, required for the appropriate recruitment of DNA repair enzymes (66). When DNA damage occurs, PARP-1 is rapidly recruited to the altered DNA and converts nicotinamide adenine dinucleotide (NAD) into ADP-ribose polymers (PAR) by attracting XRCC1, a scaffold protein which stabilizes or stimulates compounds involved in single-stranded breakage (67). PARP-1 is composed of three functional domains: the amino-terminal DNA-binding domain composed of two zinc fingers, which is necessary to bind the DNA breaks; the automodification domain, which allows the enzyme to PARate itself, and the c-terminal catalytic domain where ADP-ribose subunits are transferred from NAD+ to proteins acceptors (67). PARP inhibitors are able to block the catalytic PARP domain competing with NAD, and impairing the single-stranded DNA breakage repair activity. This mechanism induces apoptosis through the accumulation of damaged DNA in the cells. Functionally, PARP inhibitors (e.g., olaparib, talazoparib, rucaparib, and veliparib) prevent cancer cells from appropriately repair DNA damages, consequently genotoxic stress results in cancer cell death (65). Cells with BRCA1/2 wild-type can still repair the damage through HR, whereas mutated BRCA1/2 cells strictly depend on PARP activity for DNA repair. The inhibition of PARP in absence of functional BRCA genes results in *synthetic lethality*. As well-known, chemotherapy alters pathways involved in DNA damage repair; for this reason, PARP inhibitors can sensitize tumor cells to chemotherapy and radiotherapy and can induce *synthetic lethality* in tumors from patients with hereditary or sporadic mutations in BRCA1/2 genes. Different PARP inhibitors have been evaluated in preclinical studies and in clinical trials as mono or combination therapies for breast cancer patients, particularly for TNBC. In a BRCA1-deficient breast cancer mouse model, the combination of a PARP inhibitor with cisplatin or carboplatin increases the recurrence-free and overall survival, indicating that PARP inhibitors can improve the efficacy of DNA-damaging compounds (68). Moreover, Hay T. and colleagues have shown that daily treatment with olaparib for 28 days in mice with BRCA2^−/−^ mammary epithelium caused a significant regression of 46/52 tumors (69).

In clinics, different trials on breast cancer patients with BRCA1/2-defective tumors demonstrated that PARP inhibitors, such as olaparib, enhance the therapeutic response when administrated as single agents or in combination with platinum compounds.

In a first study of phase I, 60 patients, affected by different tumor types including breast cancer, were enrolled and treated with olaparib. Among these, 22 patients had BRCA1 or BRCA2 mutations. Results showed that olaparib has the capability to inhibit PARP with limited adverse effects in comparison with conventional chemotherapy, however an antitumor activity was observed only in patients carrying BRCA mutations (70). In a phase II study, two cohorts of 27 patients with confirmed BRCA1 or BRCA2 mutation-advanced breast cancer were enrolled and treated with two different doses of olaparib. The first cohort had an overall response rate (ORR) of 41% and in the second cohort an ORR of 22%; moreover no particular toxicity has been reported (71).

Furthermore, when olaparib had been combined with paclitaxel in a cohort of 19 patients with metastatic TNBC in phase I study, an ORR of about 30–40% has been observed. Particularly seven patients had a confirmed partial response and one patient remained stable with olaparib monotherapy without progression (72). To date, phase III trials are ongoing to investigate the use of olaparib in the metastatic and neoadjuvant setting, for patients with mutations in BRCA1/2 (62). Finally, a randomized phase II trial that recruited patients with TNBC and/or BRCA mutations, treated with cisplatin alone or in combination with rucaparib, showed that both treatment groups present a similar disease-free survival at 1 year follow-up (~76%), and rucaparib did not add significant toxicity to the cisplatin regimen (62, 73).

Results of clinical studies with PARP inhibitors have shown promising results in advanced breast cancer, but there is still an urgent need to identify suitable patients who may actually benefit from this treatment. Further investigations to find new strategies to efficiently impair DNA repair mechanisms in breast cancer patients could enhance the response to radio-, chemo-, and PARP inhibition therapies.

## MicroRNAs regulate DNA repair genes and radio-chemotherapy responsiveness

Alterations in DNA repair mechanisms and in miRNA expression are both features of cancer development and progression (28, 74). As reported in this review, genotoxic agents, causing DNA damage, are commonly used for radio- and chemo-therapeutic treatments in breast cancer. MiRNA up- or down-modulation is often involved in the regulation of DNA repair mechanisms (75) and it is currently known that miRNAs can regulate responsiveness to drugs (76). Thus, the alterations of miRNA expression involved in DDR mechanisms play an important role in responsiveness to radio- and chemotherapy. Recently, our group has shown that miR-302b expression in breast cancer cell lines induces cisplatin sensitivity, reducing cell viability and proliferation in response to the treatment (77). E2F1, a master regulator of the G1/S transition, is directly targeted by miR-302b. Moreover, this miRNA, through the negative regulation of E2F1, indirectly downregulates ATM, the main cellular sensor of DNA damage, affecting cell-cycle progression following cisplatin treatment. As a result, miR-302b impairs the capability to repair damaged DNA upon cisplatin treatment, enhancing apoptosis in breast cancer cells (77).

Accordingly, another group has demonstrated that miR-302 family is able to sensitize breast cancer cells to radiotherapy; in particular Liang et al. showed that the decreased expression of miR-302a induces radiotherapy resistance and the reintroduction of miR-302a expression enhances radiotherapy sensitivity in *in vitro* and *in vivo* breast cancer models, abrogating the expression of AKT1 and RAD52 (78).

Gasparini et al. revealed that miR-155 overexpression reduced RAD51 levels in human breast cancer cells, which affects the response to IR and impairs the efficiency of HR repair enhancing IR sensitivity both in *in vitro* and *in vivo* models. Moreover, a series of TNBC patients with high levels of miR-155 and low expression of RAD51 revealed a significant association with a better overall survival. Thus, miR-155 expression can be considered as a prognostic biomarker which allows to identify TNBC patients who will likely be responsive to IR-based therapeutic approach (79). It was broadly demonstrated the over-expression of the oncomiR miR-21 in tumors with relevant consequences in cell cycle, DNA damage repair, apoptosis, autophagy, and hypoxia of cancer cells during irradiation. Indeed, cell cycle progression is influenced by miR-21 through the induction of DNA damage in G2 checkpoint and the miRNA inhibition (by anti-miR-21 administration) reduced the G2/M block and induced apoptosis following radiation treatment in breast cancer (80).

Of note, the overexpression of the miR-205, an oncosuppressive miRNA, increases the response to tyrosine kinase inhibitors, lapatinib and gefitinib in preclinical breast cancer models (81). Recently, it has been described that enhanced expression of miR-205 sensitizes breast cancer cells to radiation by regulating ZEB1 and affecting DNA repair. Indeed, miR-205 directly targets Ubc13, a protein involved in the homologous recombination. Moreover, the authors demonstrated that the delivery of miR-205 mimics, by nanoliposomes in a xenograft model, has a therapeutic effect sensitizing tumor to radiation (82).

MiR-18a is upregulated in breast cancer cell lines and patient tissues, interestingly its ectopic expression downregulates ATM. This phenomenon in breast cancer cells reduced the DNA damage repair ability, the efficiency of HR and sensitized cells to radiation treatment (83). Wip1 is a regulator of DNA damage signaling pathways, in particular it inhibits the phosphorylation of some DNA repair factors, including ATM, Chk1, Chk2, p53, and others. Zhang et al have demonstrated that miR-16 targets Wip1, affecting DNA repair and sensitizing breast cancer cells to doxorubicin treatment (84). Furthermore, using a TNBC *in vivo* model, Wang et al. demonstrated that miR-96 reduces the expression of REV1 and RAD51 and consequently inhibits tumor growth after cisplatin treatment. Thus, miR-96 is a potent cisplatin sensitizer *in vivo* (85).

Finally, diverse studies have focused their attention on miRNAs directly targeting BRCA genes in breast cancer. BRCA1 and BRCA2 are tumor suppressor genes important for the HR mechanism, which is the process involved in the repair of DNA DSBs. For instance, miR-218 directly targets BRCA1 and that its restored expression in cisplatin resistant breast cancer cell lines sensitizes cells against the drug, affecting DNA damage (86). Moreover, miR-638 overexpression increases sensitivity to DNA-damaging agents, ultraviolet (UV) and cisplatin, and reduces proliferation, invasive ability, and DNA repair capabilities, by down-regulation of BRCA1 in TNBC cells (87). Table 1 summarizes miRNAs involved in the chemo- and radio-responsiveness, through the regulation of DNA repair genes, in breast cancer.

**Table 1:** miRNAs involved in the chemo- and radio-responsiveness, through the regulation of DNA repair genes.

**microRNA expression**	**Gene target**	**Drug response**
miR-302b overexpression	E2F1 and ATM	Cisplatin sensitivity (77)
miR-302a overexpression	AKT1 and RAD52	IR sensitivity (78)
miR-155 overexpression	RAD51	IR sensitivity (79)
miR-21 downregulation	G2/M block	IR sensitivity (80)
miR-205 overexpression	Ubc13	IR sensitivity (82)
miR-18a overexpression	ATM	IR sensitivity (83)
miR-16 overexpression	Wip1	Doxorubicin sensitivity (84)
miR-96 overexpression	REV1 and RAD51	Cisplatin sensitivity (85)
miR-218 overexpression	BRCA1	Cisplatin sensitivity (86)
miR-638 overexpression	BRCA1	UV and Cisplatin sensitivity (87)

## MicroRNAs regulate DNA repair genes and PARP inhibitor response

As reported above, PARP inhibitors represent one of the most innovative approaches in the development of anti-breast cancer therapies. However, whether miRNAs could influence sensitivity to PARP inhibitors has not been deeply investigated yet. Indeed, few reports have described the role of miRNAs in the modulation of PARP inhibitor response. Here, we report the main results about this regulation in breast cancer. In 2011, Moskwa et al. have demonstrated that breast cancer cells overexpressing miR-182 are more sensitive to IR and PARP inhibitors via BRCA1 targeting and impairment of DNA repair. These results were also confirmed in *in vivo* models; mice injected with breast cancer cells overexpressing miR-182 showed a reduced tumor growth when treated with PARP inhibitor olaparib (88). Moreover, it has been demonstrated that CHEK2, another gene involved in the HR, is a direct target of miR-182-5p. This regulation enhances the sensitivity to a PARP inhibitor in breast cancer cells (89). As reported by Neijenhuis et al. and Huang et al. miR-107, miR-222 and miR-103 regulate the DDR and sensitize tumor cells to PARP inhibitors in breast cancer cell lines, targeting RAD51 and impairing HR (90, 91). It is also known that miR-96 targets RAD51 and REV1, and that the overexpression of this miRNA in breast cancer in *in vitro* models results in improved sensitivity to PARP inhibitors (85). In this context, it has also been demonstrated that TGFβ regulates DNA repair genes and responsiveness to PARP inhibitors. Liu et al. have shown that two TGFβ-targeted DNA-repair genes, ATM and BRCA1, both regulated by miR-181, and MSH2 targeted by miR-21, contribute to TGFβ-induced sensitivity to PARP inhibition (92). More recently, the role of miR-664b-5p has been investigated. This miRNA is a tumor suppressor and results upregulated upon PARP inhibitor plus chemotherapy treatments. Thus miR-664b-5p has an important role in the regulation of PARP inhibitors to increase chemosensitivity by targeting CCNE2 in BRCA1 not-mutated TNBC (93). Furthermore, an interesting mechanism involving miR-151-5p and its target SMARCA5, an ISWI family member with an important role in DSB repair (94), has been proposed. Indeed, Tommasi et al. reported the possibility of considering the overexpression of PARP1 and miR-151-5p as predictive biomarkers, useful to correctly select sporadic breast cancers for treatment with PARP inhibitors.

Table 2 summarizes the miRNAs involved in the PARP inhibitor response, through the regulation of DNA repair genes, in breast cancer.

**Table 2:** miRNAs involved in the PARP inhibitors response, through the regulation of DNA repair genes.

**microRNA expression**	**Gene target**	**Drug response**
miR-182 overexpression	BRCA1	IR and PARP inhibitors sensitivity (88)
miR-182 overexpression	CHEK2	PARP inhibitors sensitivity (89)
miR-107 and miR-122 overexpression	RAD51	PARP inhibitors sensitivity (90)
miR-103 and miR-107 overexpression	RAD51 and RAD51D	PARP inhibitors sensitivity (91)
miR-96 overexpression	RAD51 and REV1	PARP inhibitors sensitivity (85)
miR-181 overexpression	ATM and BRCA1	PARP inhibitors sensitivity (92)
miR-21 overexpression	MSH2	PARP inhibitors sensitivity (92)
miR-664b-5p overexpression	CCNE2	PARP inhibitors and chemo-sensitivity (93)
miR-151-5p overexpression	SMARCA5	PARP inhibitors sensitivity (94)

## Conclusion

**Figure 2:**
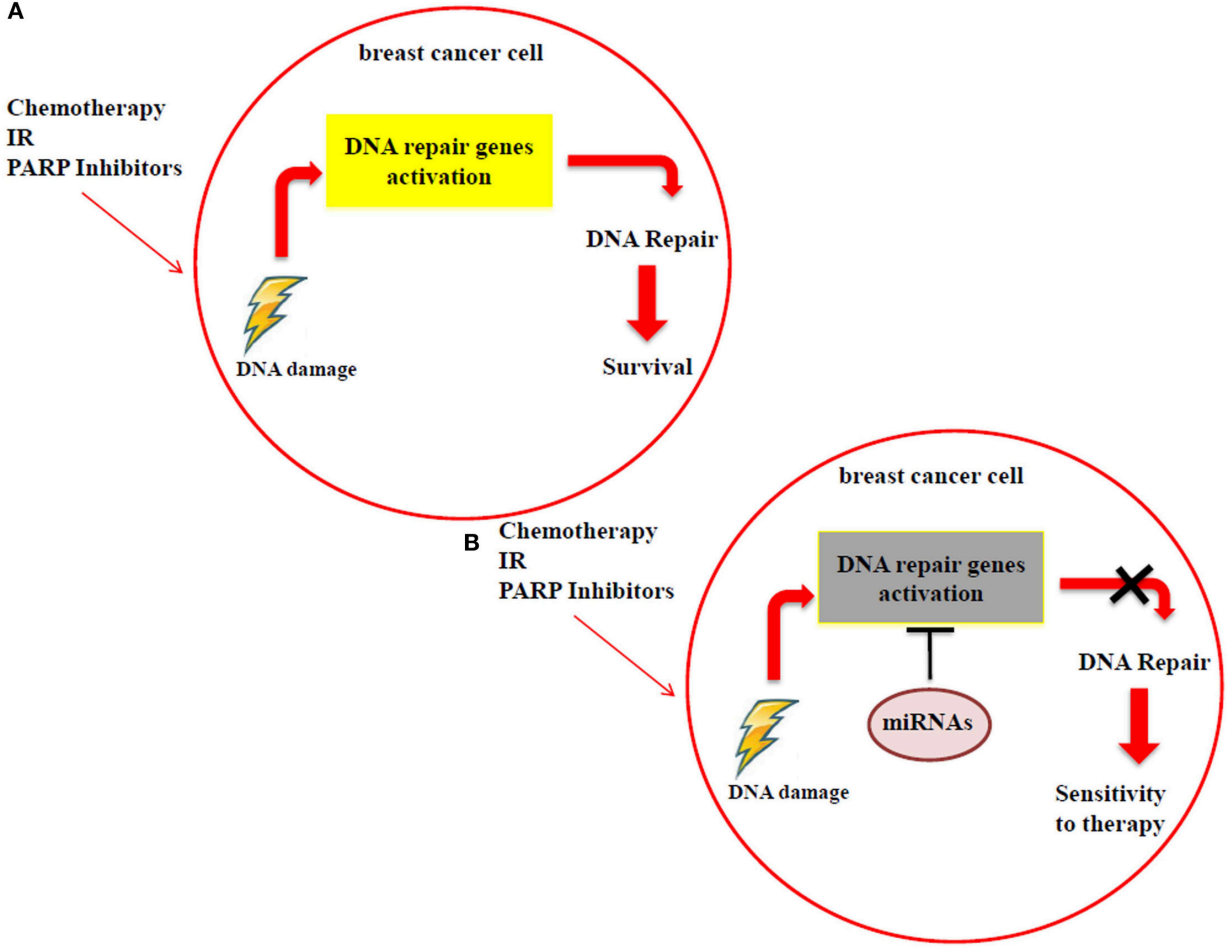
**(A)** Breast cancer cells can still repair the DNA damage, caused by radiotherapy, chemotherapy and PARP inhibitor treatments, using DDR mechanisms. **(B)** MiRNAs impair the activation of DDR mechanisms by targeting DNA repair genes, improving the sensitivity to radiotherapy, chemotherapy and PARP inhibitors.

Many studies have reported that miRNA modulation in breast cancer, by using *in vitro* and *in vivo* models, can be exploited to achieve a higher response to the DNA-damaging drugs, as radiotherapy, chemotherapy and PARP inhibitors. These small RNAs have the ability to directly target DNA repair genes, thus resulting in the impairment of DNA repair mechanisms. When breast cancer cells are treated with radiotherapy, chemotherapy or PARP inhibitors, the resulting DNA damage could be still repaired through the activation of specific DNA repair genes, such as ATM, BRCA1/2, RAD51, DNA-PK etc., thus cells survive and continue to proliferate (Figure 2A). Specific miRNAs, targeting DNA repair genes, are able to impair the mechanisms involved in repairing the DNA damage and to promote a higher sensitivity to the treatments in breast cancer cells (Figure 2B). For this reason, miRNAs could be exploited as predictive biomarkers and therapeutic tools to increase the response to DNA-damaging drugs. Considering PARP inhibitors, they are currently not in clinical practice for breast cancer and the inclusion criteria to treat patients using these drugs in clinical trials are that tumor cells are BRCA 1/2 mutated. We can speculate that the miRNAs involved in the regulation of DNA repair genes could represent a novel strategy to mimic the BRCAness phenotype, making tumor cells BRCA1/2 wild type more responsive to PARP inhibitors. However, in this context it is relevant to underline that currently the feasibility of a miRNA-based therapy in clinics has not been demonstrated yet, either alone or in combination with standard therapies. Technical and practical issues still need to be solved, such as the toxicity, off-target effects and systemic delivery. Indeed, for the clinical practice it will be necessary to identify the best strategy to deliver miRNAs directly to the tumor, i.e., by conjugation with antibodies or specific nanoparticles, thus avoiding unwanted off-target effects in healthy cells.

## Author contributions

IP, GC, and AC wrote and edited the manuscript. IP revised the new strategies of breast cancer treatment. GC revised the breast cancer and microRNAs sections. AC revised DNA damage section and the recent findings about microRNAs and DNA-damaging drug responsiveness.

### Conflict of interest statement

The authors declare that the research was conducted in the absence of any commercial or financial relationships that could be construed as a potential conflict of interest.

## Abbreviations

DDR: DNA damage response
NER: nucleotide excision repair
BER: base excision repair
MMR: mismatch repair machinery
DSBs: Double Strand Breaks
SSBs: Single-Strand Breaks
NHEJ: non-homologous end joining
HR: homologous recombination
ER: estrogen receptor
PR: progesterone receptor
HER2: epidermal growth factor receptor 2
TNBC: triple negative breast cancer
miRNAs: microRNAs
IR: ionizing radiation.

## References

[1] Lee Y Kim M Han J Yeom KH Lee S Baek SH. MicroRNA genes are transcribed by RNA polymerase II. EMBO J. (2004) 23:4051–60. 10.1038/sj.emboj.7600385 15372072 PMC524334

[2] Gregory RI Yan KP Amuthan G Chendrimada T Doratotaj B Cooch N. The Microprocessor complex mediates the genesis of microRNAs. Nature (2004) 432:235–40. 10.1038/nature03120 15531877

[3] Park JE Heo I Tian Y Simanshu DK Chang H Jee D. Dicer recognizes the 5' end of RNA for efficient and accurate processing. Nature (2011) 475:201–5. 10.1038/nature10198 21753850 PMC4693635

[4] Chendrimada TP Gregory RI Kumaraswamy E Norman J Cooch N Nishikura K. TRBP recruits the Dicer complex to Ago2 for microRNA processing and gene silencing. Nature (2005) 436:740–4. 10.1038/nature03868 15973356 PMC2944926

[5] Krol J Loedige I Filipowicz W. The widespread regulation of microRNA biogenesis, function and decay. Nat Rev Genet. (2010) 11:597–610. 10.1038/nrg2843 20661255

[6] Carthew RW Sontheimer EJ. Origins and Mechanisms of miRNAs and siRNAs. Cell (2009) 136:642–55. 10.1016/j.cell.2009.01.035 19239886 PMC2675692

[7] Lee RC Feinbaum RL Ambros V The C. elegans heterochronic gene lin-4 encodes small RNAs with antisense complementarity to lin-14. Cell (1993) 75:843–854. 10.1016/0092-8674(93)90529-Y 8252621

[8] Han C Wan G Langley RR Zhang X Lu X. Crosstalk between the DNA damage response pathway and microRNAs. Cell Mol Life Sci. (2012) 69:2895–906. 10.1007/s00018-012-0959-8 22430204 PMC11115143

[9] Iorio MV Croce CM. Causes and consequences of microRNA dysregulation. Cancer J. (2012) 18:215–22. 10.1097/PPO.0b013e318250c001 22647357 PMC3528102

[10] Kaboli PJ Rahmat A Ismail P Ling KH. MicroRNA-based therapy and breast cancer: a comprehensive review of novel therapeutic strategies from diagnosis to treatment. Pharmacol Res. (2015) 97:104–21. 10.1016/j.phrs.2015.04.015 25958353

[11] Rupaimoole R Slack FJ. MicroRNA therapeutics: towards a new era for the management of cancer and other diseases. Nat Rev Drug Discov. (2017) 16:203–22. 10.1038/nrd.2016.246 28209991

[12] Catela Ivkovic T Voss G Cornella H Ceder Y. microRNAs as cancer therapeutics: a step closer to clinical application. Cancer Lett. (2017) 407:113–22. 10.1016/j.canlet.2017.04.007 28412239

[13] Torre LA Bray F Siegel RL Ferlay J Lortet-Tieulent J Jemal A. Global cancer statistics, 2012. CA Cancer J Clin. (2015) 65:87–108. 10.3322/caac.21262 25651787

[14] Turashvili G Brogi E. Tumor heterogeneity in breast cancer. Front Med. (2017) 4:227. 10.3389/fmed.2017.00227 29276709 PMC5727049

[15] Hammond ME Hayes DF Wolff AC Mangu PB Temin S. American society of Clinical Oncology/College of American Pathologists guideline recommendations for immunohistochemical testing of estrogen and progesterone receptors in breast cancer. J Oncol Pract. (2010) 6:195–7. 10.1200/JOP.777003 21037871 PMC2900870

[16] Wolff AC Hammond ME Hicks DG Dowsett M McShane LM Allison KH. Recommendations for human epidermal growth factor receptor 2 testing in breast cancer: American Society of Clinical Oncology/College of American Pathologists clinical practice guideline update. J Clin Oncol. (2013) 31:3997–4013. 10.1200/JCO.2013.50.9984 24101045

[17] Perou CM Sorlie T Eisen MB van de Rijn M Jeffrey SS Rees CA. Molecular portraits of human breast tumours. Nature (2000) 406:747–52. 10.1038/35021093 10963602

[18] Sorlie T Perou CM Tibshirani R Aas T Geisler S Johnsen H. Gene expression patterns of breast carcinomas distinguish tumor subclasses with clinical implications. Proc Natl Acad Sci USA. (2001) 98:10869–74. 10.1073/pnas.191367098 11553815 PMC58566

[19] Sorlie T Wang Y Xiao C Johnsen H Naume B Samaha RR. Distinct molecular mechanisms underlying clinically relevant subtypes of breast cancer: gene expression analyses across three different platforms. BMC Genomics (2006) 7:127. 10.1186/1471-2164-7-127 16729877 PMC1489944

[20] Majidinia M Yousefi B. DNA repair and damage pathways in breast cancer development and therapy. DNA Repair (2017) 54:22–9. 10.1016/j.dnarep.2017.03.009 28437752

[21] Herschkowitz JI Simin K Weigman VJ Mikaelian I Usary J Hu Z. Identification of conserved gene expression features between murine mammary carcinoma models and human breast tumors. Genome Biol. (2007) 8:R76. 10.1186/gb-2007-8-5-r76 17493263 PMC1929138

[22] Prat A Perou CM. Deconstructing the molecular portraits of breast cancer. Mol Oncol. (2011) 5:5–23. 10.1016/j.molonc.2010.11.003 21147047 PMC5528267

[23] Lehmann BD Bauer JA Chen X Sanders ME Chakravarthy AB Shyr Y. Identification of human triple-negative breast cancer subtypes and preclinical models for selection of targeted therapies. J Clin Invest. (2011) 121:2750–67. 10.1172/JCI45014 21633166 PMC3127435

[24] The Cancer Genome Atlas Network Comprehensive molecular portraits of human breast tumours. Nature (2012) 490:61–70. 10.1038/nature11412 23000897 PMC3465532

[25] Curtis C Shah SP Chin SF Turashvili G Rueda OM Dunning MJ. The genomic and transcriptomic architecture of 2,000 breast tumours reveals novel subgroups. Nature (2012) 486:346–52. 10.1038/nature10983 22522925 PMC3440846

[26] Prat A Pineda E Adamo B Galvan P Fernandez A Gaba L. Clinical implications of the intrinsic molecular subtypes of breast cancer. Breast (2015) 24(Suppl. 2):S26–35. 10.1016/j.breast.2015.07.008 26253814

[27] Iorio MV Ferracin M Liu CG Veronese A Spizzo R Sabbioni S. MicroRNA gene expression deregulation in human breast cancer. Cancer Res. (2005) 65:7065–70. 10.1158/0008-5472.CAN-05-1783 16103053

[28] Kumar MS Lu J Mercer KL Golub TR Jacks T. Impaired microRNA processing enhances cellular transformation and tumorigenesis. Nat Genet. (2007) 39:673–7. 10.1038/ng2003 17401365

[29] Ma L Young J Prabhala H Pan E Mestdagh P Muth D. miR-9, a MYC/MYCN-activated microRNA, regulates E-cadherin and cancer metastasis. Nat Cell Biol. (2010) 12:247–56. 10.1038/ncb2024 20173740 PMC2845545

[30] Ma L Teruya-Feldstein J Weinberg RA. Tumour invasion and metastasis initiated by microRNA-10b in breast cancer. Nature (2007) 449:682–8. 10.1038/nature06174 17898713

[31] Coulombe G Tyldesley S Speers C Paltiel C quino-Parsons C Bernstein V. Is mastectomy superior to breast-conserving treatment for young women? Int J Radiat Oncol Biol Phys. (2007) 67:1282–90. 10.1016/j.ijrobp.2006.11.032 17275207

[32] Scott GK Goga A Bhaumik D Berger CE Sullivan CS Benz CC Coordinate suppression of ERBB2 and ERBB3 by enforced expression of micro-RNA miR-125a or miR-125b. J Biol Chem. (2007) 282:1479–86. 10.1074/jbc.M609383200 17110380

[33] D'Ippolito E Iorio MV. MicroRNAs and triple negative breast cancer. Int J Mol Sci. (2013) 14:22202–20. 10.3390/ijms141122202 24284394 PMC3856060

[34] Hanahan D Weinberg RA. Hallmarks of cancer: the next generation. Cell (2011) 144:646–74. 10.1016/j.cell.2011.02.013 21376230

[35] Hoeijmakers JH. Genome maintenance mechanisms for preventing cancer. Nature (2001) 411:366–74. 10.1038/35077232 11357144

[36] Natarajan AT Palitti F. DNA repair and chromosomal alterations. Mutat Res. (2008) 657:3–7. 10.1016/j.mrgentox.2008.08.017 18801460

[37] Tessitore A Cicciarelli G Del VF Gaggiano A Verzella D Fischietti M. MicroRNAs in the DNA damage/repair network and cancer. Int J Genomics (2014) 2014:820248. 10.1155/2014/820248 24616890 PMC3926391

[38] Acharya S Foster PL Brooks P Fishel R. The coordinated functions of the E. coli MutS and MutL proteins in mismatch repair. Mol Cell. (2003) 12:233–46. 10.1016/S1097-2765(03)00219-3 12887908

[39] Kolodner RD Mendillo ML Putnam CD. Coupling distant sites in DNA during DNA mismatch repair. Proc Natl Acad Sci USA. (2007) 104:12953–54. 10.1073/pnas.0705698104 17664420 PMC1941800

[40] Lord CJ Ashworth A. The DNA damage response and cancer therapy. Nature (2012) 481:287–94. 10.1038/nature10760 22258607

[41] O'Connor MJ. Targeting the DNA damage response in cancer. Mol Cell (2015) 60:547–60. 10.1016/j.molcel.2015.10.040 26590714

[42] Ciccia A Elledge SJ. The DNA damage response: making it safe to play with knives. Mol Cell (2010) 40:179–204. 10.1016/j.molcel.2010.09.019 20965415 PMC2988877

[43] Michelini F Pitchiaya S Vitelli V Sharma S Gioia U Pessina F. Damage-induced lncRNAs control the DNA damage response through interaction with DDRNAs at individual double-strand breaks. Nat Cell Biol. (2017) 19:1400–11. 10.1038/ncb3643 29180822 PMC5714282

[44] Matsuoka S Ballif BA Smogorzewska A McDonald ER III Hurov KE Luo J. ATM and ATR substrate analysis reveals extensive protein networks responsive to DNA damage. Science (2007) 316:1160–6. 10.1126/science.1140321 17525332

[45] Kastan MB Lim DS. The many substrates and functions of ATM. Nat Rev Mol Cell Biol. (2000) 1:179–86. 10.1038/35043058 11252893

[46] Abraham RT. Cell cycle checkpoint signaling through the ATM and ATR kinases. Genes Dev. (2001) 15:2177–96. 10.1101/gad.914401 11544175

[47] Shiloh Y. ATM and ATR: networking cellular responses to DNA damage. Curr Opin Genet Dev. (2001) 11:71–7. 10.1016/S0959-437X(00)00159-3 11163154

[48] Bartek J Lukas J DNA damage checkpoints: from initiation to recovery or adaptation. Curr Opin Cell Biol. (2007) 19:238–45. 10.1016/j.ceb.2007.02.009 17303408

[49] Lieber MR. The mechanism of double-strand DNA break repair by the nonhomologous DNA end-joining pathway. Annu Rev Biochem. (2010) 79; 181–211. 10.1146/annurev.biochem.052308.093131 20192759 PMC3079308

[50] Shibata A Conrad S Birraux J Geuting V Barton O Ismail A. Factors determining DNA double-strand break repair pathway choice in G2 phase. EMBO J. (2011) 30:1079–92. 10.1038/emboj.2011.27 21317870 PMC3061033

[51] Bottai G Pasculli B Calin GA Santarpia L. Targeting the microRNA-regulating DNA damage/repair pathways in cancer. Expert Opin Biol Ther. (2014) 14:1667–83. 10.1517/14712598.2014.950650 25190496

[52] Harris LN Ismaila N McShane LM Andre F Collyar DE Gonzalez-Angulo AM. Use of biomarkers to guide decisions on adjuvant systemic therapy for women with early-stage invasive breast cancer: American Society of Clinical Oncology clinical practice guideline. J Clin Oncol. (2016) 34:1134–50. 10.1200/JCO.2015.65.2289 26858339 PMC4933134

[53] Saha P Nanda R. Concepts and targets in triple-negative breast cancer: recent results and clinical implications. Ther Adv Med Oncol. (2016) 8:351–9. 10.1177/1758834016657071 27583027 PMC4981296

[54] Sjostedt S Bezak E. Non-targeted effects of ionising radiation and radiotherapy. Australas Phys Eng Sci Med. (2010) 33:219–31. 10.1007/s13246-010-0030-8 20857259

[55] Kadhim M Salomaa S Wright E Hildebrandt G Belyakov OV Prise KM. Non-targeted effects of ionising radiation–implications for low dose risk. Mutat Res. (2013) 752:84–98. 10.1016/j.mrrev.2012.12.001 23262375 PMC4091999

[56] Stankevicins L meida da Silva AP Ventura Dos Passos F Dos Santos Ferreira E Menks Ribeiro MC David G. MiR-34a is up-regulated in response to low dose, low energy X-ray induced DNA damage in breast cells. Radiat Oncol. (2013) 8:231–8. 10.1186/1748-717X-8-231 24094113 PMC3829672

[57] Shimura T. Acquired radioresistance of cancer and the AKT/GSK3beta/cyclin D1 overexpression cycle. J Radiat Res. (2011) 52:539–44. 10.1269/jrr.11098 21881296

[58] Seton-Rogers S. Chemotherapy: preventing competitive release. Nat Rev Cancer (2016) 16:199. 10.1038/nrc.2016.28 27009390

[59] Pan ST Li ZL He ZX Qiu JX Zhou SF. Molecular mechanisms for tumour resistance to chemotherapy. Clin Exp Pharmacol Physiol. (2016) 43:723–37. 10.1111/1440-1681.12581 27097837

[60] Garofalo M Croce CM. MicroRNAs as therapeutic targets in chemoresistance. Drug Resist Updat. (2013) 16:47–59. 10.1016/j.drup.2013.05.001 23757365 PMC3858390

[61] Shah SP Roth A Goya R Oloumi A Ha G Zhao Y. The clonal and mutational evolution spectrum of primary triple-negative breast cancers. Nature (2012) 486:395–9. 10.1038/nature10933 22495314 PMC3863681

[62] Bianchini G Balko JM Mayer IA Sanders ME Gianni L. Triple-negative breast cancer: challenges and opportunities of a heterogeneous disease. Nat Rev Clin Oncol. (2016) 13:674–90. 10.1038/nrclinonc.2016.66 27184417 PMC5461122

[63] Lord CJ Ashworth A. BRCAness revisited. Nat Rev Cancer (2016) 16:110–20. 10.1038/nrc.2015.21 26775620

[64] Crown J O'Shaughnessy J Gullo G. Emerging targeted therapies in triple-negative breast cancer. Ann Oncol. (2012) 23(Suppl. 6):vi56–65. 10.1093/annonc/mds196 23012305

[65] Ohmoto A Yachida S. Current status of poly(ADP-ribose) polymerase inhibitors and future directions. Onco Targets Ther. (2017) 10:5195–208. 10.2147/OTT.S139336 29138572 PMC5667784

[66] Rajiah IR Skepper J. Differential localisation of PARP-1 N-terminal fragment in PARP-1(+/+) and PARP-1(-/-) murine cells. Mol Cells (2014) 37:526–31. 10.14348/molcells.2014.0077 25078451 PMC4132304

[67] Rouleau M Patel A Hendzel MJ Kaufmann SH Poirier GG. PARP inhibition: PARP1 and beyond. Nat Rev Cancer (2010) 10:293–301. 10.1038/nrc2812 20200537 PMC2910902

[68] Rottenberg S Jaspers JE Kersbergen A van der BE Nygren AO Zander SA. High sensitivity of BRCA1-deficient mammary tumors to the PARP inhibitor AZD2281 alone and in combination with platinum drugs. Proc Natl Acad Sci USA. (2008) 105:17079–84. 10.1073/pnas.0806092105 18971340 PMC2579381

[69] Hay T Matthews JR Pietzka L Lau A Cranston A Nygren AO. Poly(ADP-ribose) polymerase-1 inhibitor treatment regresses autochthonous Brca2/p53-mutant mammary tumors *in vivo* and delays tumor relapse in combination with carboplatin. Cancer Res. (2009) 69:3850–5. 10.1158/0008-5472.CAN-08-2388 19383921

[70] Fong PC Boss DS Yap TA Tutt A Wu P Mergui-Roelvink M. Inhibition of poly(ADP-ribose) polymerase in tumors from BRCA mutation carriers. N Engl J Med. (2009) 361:123–34. 10.1056/NEJMoa0900212 19553641

[71] Tutt A Robson M Garber JE Domchek SM Audeh MW Weitzel JN Oral poly(ADP-ribose) polymerase inhibitor olaparib in patients with BRCA1 or BRCA2 mutations and advanced breast cancer: a proof-of-concept trial. Lancet (2010) 376:235–44. 10.1016/S0140-6736(10)60892-6 20609467

[72] Dent RA Lindeman GJ Clemons M Wildiers H Chan A McCarthy NJ. Phase I trial of the oral PARP inhibitor olaparib in combination with paclitaxel for first- or second-line treatment of patients with metastatic triple-negative breast cancer. Breast Cancer Res. (2013) 15:R88. 10.1186/bcr3484 24063698 PMC3979135

[73] Dwadasi S Tong Y Walsh T Danso MA Ma CX Silverman P Cisplatin with or without rucaparib after preoperative chemotherapy in patients with triple-negative breast cancer (TNBC): Hoosier Oncology Group BRE09 (abstract). J.Clin.Oncol. (2014) 32:1019.

[74] Bartkova J Horejsi Z Koed K Kramer A Tort F Zieger K. DNA damage response as a candidate anti-cancer barrier in early human tumorigenesis. Nature (2005) 434:864–70. 10.1038/nature03482 15829956

[75] Wang Y Taniguchi T. MicroRNAs and DNA damage response: implications for cancer therapy. Cell Cycle (2013) 12:32–42. 10.4161/cc.23051 23255103 PMC3570514

[76] Iorio MV Croce CM. MicroRNA dysregulation in cancer: diagnostics, monitoring and therapeutics. A comprehensive review. EMBO Mol Med. (2012) 4:143–59. 10.1002/emmm.201100209 22351564 PMC3376845

[77] Cataldo A Cheung DG Balsari A Tagliabue E Coppola V Iorio MV. miR-302b enhances breast cancer cell sensitivity to cisplatin by regulating E2F1 and the cellular DNA damage response. Oncotarget (2016) 7:786–97. 10.18632/oncotarget.6381 26623722 PMC4808033

[78] Liang Z Ahn J Guo D Votaw JR Shim H. MicroRNA-302 replacement therapy sensitizes breast cancer cells to ionizing radiation. Pharm Res. (2013) 30:1008–16. 10.1007/s11095-012-0936-9 23184229 PMC3594086

[79] Gasparini P Lovat F Fassan M Casadei L Cascione L Jacob NK. Protective role of miR-155 in breast cancer through RAD51 targeting impairs homologous recombination after irradiation. Proc Natl Acad Sci USA. (2014) 111:4536–41. 10.1073/pnas.1402604111 24616504 PMC3970505

[80] Anastasov N Hofig I Vasconcellos IG Rappl K Braselmann H Ludyga N. Radiation resistance due to high expression of miR-21 and G2/M checkpoint arrest in breast cancer cells. Radiat Oncol. (2012) 7:206–7. 10.1186/1748-717X-7-206 23216894 PMC3573984

[81] Iorio MV Casalini P Piovan C Di Leva G Merlo A Triulzi T. microRNA-205 regulates HER3 in human breast cancer. Cancer Res. (2009) 69:2195–200. 10.1158/0008-5472.CAN-08-2920 19276373

[82] Zhang P Wang L Rodriguez-Aguayo C Yuan Y Debeb BG Chen D. miR-205 acts as a tumour radiosensitizer by targeting ZEB1 and Ubc13. Nat Commun. (2014) 5:5671. 10.1038/ncomms6671 25476932 PMC4377070

[83] Song L Lin C Wu Z Gong H Zeng Y Wu J. miR-18a impairs DNA damage response through downregulation of ataxia telangiectasia mutated (ATM) kinase. PLoS ONE (2011) 6:e25454. 10.1371/journal.pone.0025454 21980462 PMC3181320

[84] Zhang X Wan G Mlotshwa S Vance V Berger FG Chen H. Oncogenic Wip1 phosphatase is inhibited by miR-16 in the DNA damage signaling pathway. Cancer Res. (2010) 70:7176–86. 10.1158/0008-5472.CAN-10-0697 20668064 PMC2940956

[85] Wang Y Huang JW Calses P Kemp CJ Taniguchi T. MiR-96 downregulates REV1 and RAD51 to promote cellular sensitivity to cisplatin and PARP inhibition. Cancer Res. (2012) 72:4037–46. 10.1158/0008-5472.CAN-12-0103 22761336 PMC3421071

[86] He X Xiao X Dong L Wan N Zhou Z Deng H. MiR-218 regulates cisplatin chemosensitivity in breast cancer by targeting BRCA1. Tumour Biol. (2015) 36:2065–75. 10.1007/s13277-014-2814-z 25394901

[87] Tan X Peng J Fu Y An S Rezaei K Tabbara S. miR-638 mediated regulation of BRCA1 affects DNA repair and sensitivity to UV and cisplatin in triple-negative breast cancer. Breast Cancer Res. (2014) 16:435. 10.1186/s13058-014-0435-5 25228385 PMC4303116

[88] Moskwa P Buffa FM Pan Y Panchakshari R Gottipati P Muschel RJ. miR-182-mediated downregulation of BRCA1 impacts DNA repair and sensitivity to PARP inhibitors. Mol Cell (2011) 41:210–20. 10.1016/j.molcel.2010.12.005 21195000 PMC3249932

[89] Krishnan K Steptoe AL Martin HC Wani S Nones K Waddell N. MicroRNA-182-5p targets a network of genes involved in DNA repair. RNA (2013) 19:230–42. 10.1261/rna.034926.112 23249749 PMC3543090

[90] Neijenhuis S Bajrami I Miller R Lord CJ Ashworth A. Identification of miRNA modulators to PARP inhibitor response. DNA Repair (2013) 12:394–402. 10.1016/j.dnarep.2013.02.003 23570906

[91] Huang JW Wang Y Dhillon KK Calses P Villegas E Mitchell PS. Systematic screen identifies miRNAs that target RAD51 and RAD51D to enhance chemosensitivity. Mol Cancer Res. (2013) 11:1564–73. 10.1158/1541-7786.MCR-13-0292 24088786 PMC3869885

[92] Liu L Zhou W Cheng CT Ren X Somlo G Fong MY. TGFbeta induces “BRCAness” and sensitivity to PARP inhibition in breast cancer by regulating DNA-repair genes. Mol Cancer Res. (2014) 12:1597–609. 10.1158/1541-7786.MCR-14-0201 25103497 PMC4233161

[93] Song W Tang L Xu Y Xu J Zhang W Xie H PARP inhibitor increases chemosensitivity by upregulating miR-664b-5p in BRCA1-mutated triple-negative breast cancer. Sci Rep. (2017) 7:42319 10.1038/srep42319 28176879 PMC5296748

[94] Tommasi S Pinto R Danza K Pilato B Palumbo O Micale L. miR-151-5p, targeting chromatin remodeler SMARCA5, as a marker for the BRCAness phenotype. Oncotarget (2016) 7:80363–72. 10.18632/oncotarget.10345 27385001 PMC5348325

